# Habitat effects on intra‐species variation in functional morphology: Evidence from freshwater fish

**DOI:** 10.1002/ece3.4555

**Published:** 2018-10-25

**Authors:** Fangmin Shuai, Shixiao Yu, Sovan Lek, Xinhui Li

**Affiliations:** ^1^ Pearl River Fisheries Research Institute CAFS Guangzhou China; ^2^ Ministry of Agriculture Experimental Station for Scientific Observation on Fishery Resources and Environment in the Middle and Lower Reaches of Pearl River Guangdong China; ^3^ State Key Laboratory of Biocontrol Department of Ecology School of Life Sciences Sun Yat‐sen University Guangzhou China; ^4^ Université de Toulouse ‐ Paul Sabatier Toulouse Cedex France

**Keywords:** altitude, freshwater fish, functional morphology, habitat

## Abstract

Biotic‐environment interactions have long been considered an important factor in functional phenotype differentiation in organisms. The differentiation processes determining functional phenotypes can reveal important mechanisms yielding differences in specific functions of animal traits in the ecosystem. In the present study, we examined functional morphological variations in relation to increasing geographic altitude. Six fish species were examined for how environment factors affect intra‐specific functional morphology in the subtropical Pearl River in southern China. Functional morphology traits revealed variable effects due to geographic elevation, although spatial autocorrelation existed among the species tested. The results showed that high‐elevation individuals had a more narrow‐bodied morphology, with more flexible maneuvrability when swimming, and more evenly distributed musculature than low‐elevation individuals. Low‐elevation individuals preyed upon larger food sources than high‐elevation individuals in some species. Fish functional morphology was strongly affected by regional environmental factors (such as elevation and water temperature) and physical characteristics of local rivers (such as flow velocity, river fractals, and coefficients of fluvial facies). In addition, the effects of the regional factors were stronger than those of the local factors in the Pearl River. Furthermore, it was found that morphological traits associated with locomotion were primarily effected by the river's physical characteristics. While morphological traits associated with food acquisition were primarily affected by water chemical factors (such as DO, water clarity, NH_4_‐N concentration, and TDS). These results demonstrated that habitat has an influence on the biological morphology of fish species, which further affects the functioning of the organism within the ecosystem.

## 1 INTRODUCTION

The aim of eco‐morphological research was to understand the responses of organisms’ morphological characteristics to their habitat characteristics across individuals, populations, and species (Motta, Norton, & Luczkovich, 1995; Wainwright, 1991; Wikramanayake, 1990). Several inter‐species studies across a variety of terrestrial and aquatic species have demonstrated a close correlation between the external morphology of an organism and its function in the ecosystem (Binning & Chapman, 2010; Villéger, Miranda, Hernández, & Mouillot, 2010). It is becoming accepted that a focus on functional morphology as it relates to environmental gradients could be used to identify general patterns of variation and make better predictions of the responses of natural communities to environmental changes (Olden, Jackson, & Peres‐Neto, 2002; Pease, González‐Díaz, Rodiles‐Hernández, & Winemiller, 2012).

Intra‐specific variation in functional morphological adaptations to different habitats can provide exciting insights into the functional significance of phenotypic traits (Chapman et al., 2015). For example, Darwin's finches (Grant, 1999), neotropical bats (Swartz, Freeman, & Stockwell, 2003), and Caribbean labrid fishes (Hulsey & De Leon, 2005), represent distinct species that possess striking morphological specializations in their feeding behaviors. Unique traits are often well adapted to the unique ecological function, such as food acquisition. The results of early studies indicated that morphological variations reflect natural selection for locally adaptive traits (Gatz, 1979). However, it has subsequently been shown that morphological differences can result from phenotypic plasticity, where habitat variables directly influence the phenotype of an organism (Bears, Drever, & Martin, 2008; Pigliucci, 2005).

Another group of species that has been well studied is freshwater fish (Chapman et al., 2015). Freshwater fish use a rich diversity of habitats and with high plasticity in body morphology, it has been considered the best animal model to study the relationship between morphological changes and environment gradients (Svanbäck & Eklöv, 2006). Since Liem (1980) first reported that morphological specialists were closely related to dietary generalists among fish species, follow‐up studies have shown that aquatic medium has provided opportunities for fish species to establish a range of feeding repertoires to exploit their prey, which has subsequently enabled them to develop a more versatile feeding morphology than other vertebrates (Binning & Chapman, 2010). Later studies also revealed that several ecological characteristics of freshwater fish species are linked with morphology variations in the bodies of freshwater fish species (Blanck, Tedesco, & Lamouroux, 2007; Gatz, 1979; Webb, 1984). These characteristics mainly include predation (Brönmark & Miner, 1992) and habitat use (Leal, Junqueira, & Pompeu, 2011). For example, intestinal length is closely related to the degree of herbivory (Elliott & Bellwood, 2003). Mouth gape has also been shown to be closely linked with prey size and the degree of piscivory. The relative orientation of the mouth indicates the depth at which feeding typically occurs, or of the position of the predator in relation to its prey (Davis, Pusey, & Pearson, 2012).

Several other studies have described a strong correlation between morphology and locomotion among fish species. For example, fish that occupied high flow habitats were found to have a significantly more streamlined body shape than fish occupying low flow habitats (Chapman et al., 2015; Collin & Fumagalli, 2011). Strong correlations were also shown to exist between stream hydraulics and body shape of fishes (Lamouroux, Poff, & Angermeier, 2002). For example, fish can reduce the cost of drag and energy losses in turbulent currents by evolving a narrow and more streamlined body shape, which enables them to swim in a steadier manner (Chapman et al., 2015; Langerhans & Reznick, 2010; Webb, 1984).

Although a number of studies have been conducted to investigate the mechanisms controlling variations in inter‐species functional morphology, the operation of these mechanisms in intra‐species functional morphology remains poorly understood (Binning & Chapman, 2010). There is no general consensus regarding the operation of the mechanisms leading to spatial variations in intra‐specific functional morphology. Understanding the intra‐specific variation in functional morphology first requires an understanding of the response of organisms to environmental challenges across populations and species (Motta et al., 1995).

The purpose of this study was to investigate how regional and local habitats affect intra‐species morphology variation by analyzing the relationship between functional morphology and environment gradients in the large subtropical Pearl River in southern China. Morphological traits linking food acquisition and locomotion (Villéger et al., 2010; Villégier, Mason, & Mouillot, 2008) were measured in our study. Fish were obtained from three geographical locations along the stream ranging from high altitude to medium, to low‐altitude streams. A key innovative feature of this study is that we analyzed six fish species with different ecological characteristics (two piscivore species, two omnivore species, and two herbivore species). The Pearl River was chosen as the study area because there are significant differences between upper and lower streams. Studies such as these have rarely been performed, despite their ecological importance. The present study allowed for prediction of how patterns of functional morphology of fish respond to the patterns of habitat gradients.

## 2 MATERIALS AND METHODS

### 2.1 Study area

**Figure 1:**
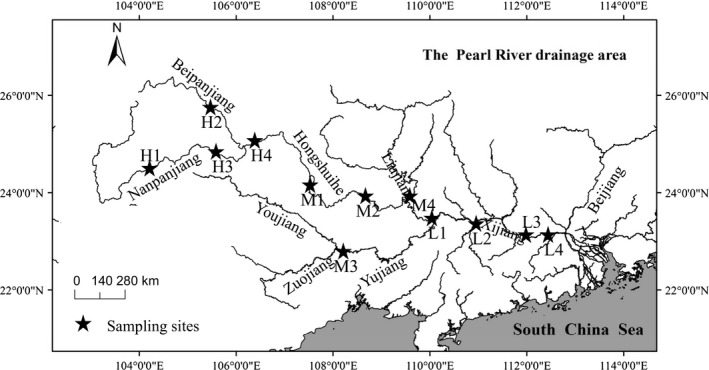
Geographic location of 12 sampling sites along the Pearl River

This study was conducted in the longest subtropical Pearl River in southern China, which originates from the Maxiong Mountain (with an altitude of 2,444 m), and ultimately flows into the South China Sea, stretching some 2,400 km. It has been characterized as having rich aquatic biological resources due to the mild climate in this region and abundant food supply, supporting more than 380 fish species (Shuai, Li, Chen, Li, & Lek, 2017). A total of 12 sampling sites comprising three groups (high, medium, and low altitude) were established to provide a comparison of intra‐specific variations in functional morphology of freshwater fish (Table 1, Figure 1).

**Table 1:** Coordinates of the 12 sampling locations along the Pearl River basin

Group	Sites	Name	Coordinates	Altitude(m)
H	H1	Luoping	104°1′47″E, 25°25′17″N	825
H	H2	Zhenfeng	107°59′17E”, 24°44′5″N	364
H	H3	Ceheng	105°47′953″E, 24°42′17″N	360
H	H4	Tiane	108°52′22″E, 23°48′43″N	336
M	M1	Dahua	107°59′16″E, 23°44′5″N	143
M	M2	Hesan	110°04′19″E, 23°24′16″N	81
M	M3	Nanning	108°19′11″E, 22°49′12″N	79
M	M4	Shilong	109°42′03″E, 24°33′12″N	73
L	L1	Guiping	110°53′6″E, 23°21′46″N	23
L	L2	Tengxian	112°27′33″E, 23°4′54″N	17
L	L3	Deqing	111°46′33″E, 23°8′36″N	11
L	L4	Zhaoqing	110°04′20″E, 23°24′15″N	9

Note

H: high‐altitude group;L: low‐altitude group; M: medium altitude group.

### 2.2 Study species

In this study, six fish species (two piscivore, two omnivore, and two herbivore species, which can be commonly found in each plot and have differences in ecological characteristics) were selected to investigate how divergent habitats affect intra‐species morphology variation in the Pearl River. Scientific names and ecological habitats of each species are shown in Table 2.

**Table 2:** List of fish species and their ecological characteristics in the Pearl River

Species	Trophic guild	Ecological habits
Perciformes		
Serranidae		
*Siniperca kneri*	Piscivore	Demersal fish
Siluriformes		
Bagridae		
*Mystus guttatus*	Piscivore	Demersal and sedentary fish
Cypriniformes		
Cyprinidae		
*Cyprinus carpio*	Omnivore	Demersal fish
*Carassius auratus*	Omnivore	Pelagic fish
*Onychosotoma gerlachi*	Herbivore	Demersal fish
*Osteochilus salsburyi*	Herbivore	Pelagic fish

### 2.3 Data collection

#### 2.3.1 Fish samples

**Figure 2:**
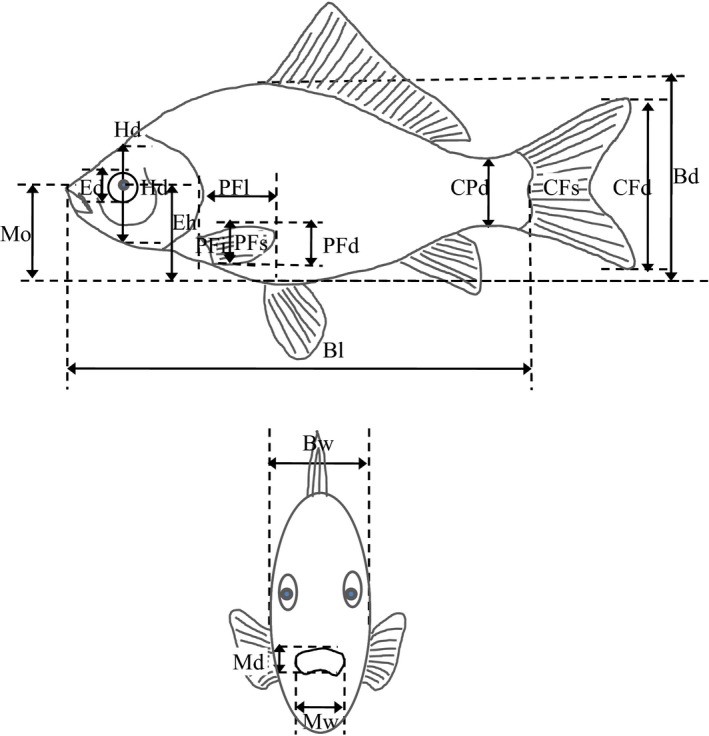
Measurement of external morphology traits

Fish samples were collected seasonally at each sampling site from 2015 to 2016. *Mystus guttatus* communities were sampled by using traditional fishing hooks. The remaining five species communities were sampled with gillnets (length: 10 m, height: 2.5 m; mesh‐size: 20 mm) and cast nets (height: 5 m, diameter: 5 m; mesh‐size: 40 mm). One sampling site being sampled per day and lasted 10 hr. Fish that were captured were immediately identified and photographed. Morphological characteristics of species were measured directly using a digital calliper and through photograph (ImageJ; Figure 2). Many studies have demonstrated that different life‐stages within a species can have different functional traits (Miller & Rudolf, 2011; Rudolf & Rasmussen, 2013; Zhao, Villéger, Lek, & Cucherousset, 2014). Therefore, in this study, for all individuals, only adulthood and non‐pregnant females were measured. Morphological traits were measured on a minimum of 20 adult individuals for each species in the study site.

Because important survival functions such as food acquisition and locomotion usually involve coordinated use of multiple organs simultaneously (Arreola & Westneat, 1996; Mouillot, Graham, Villéger, Mason, & Bellwood, 2013), a total of 19 measurements describing the morphological traits and mirroring vital functions performed by fish were made in this study (Villégier et al., 2008; Villéger et al., 2010; Albouy et al., 2011; Zhao et al., 2014; Figure 2, Table 3). These attributes, such as oral gape surface, gill raker length, gut length, and eye size, are all involved in food acquisition. Similarly, body compression index, body section area, pectoral fin shape, and fin area are all involved in locomotion (Villéger et al., 2010). Measurements were then converted into eight complementary functional morphological traits that were closely related to food acquisition and locomotion (Table 4).

**Table 3:** List of the 19 measurements (adapted from Villéger et al., 2010)

Code	Measurement	Code	Measurement
Bd	Body depth	Hd	Head depth along the vertical axis of the eye
Bl	Body standard length	M	Body weight
Bw	Body width	Md	Mouth depth
CFd	Caudal fin depth	Mo	Distance from the top of the mouth to the bottom of the head
CFs	Caudal fin surface	Mw	Mouth width
CPd	Caudal peduncle minimal depth	PFd	Body depth at the level of the pectoral fin insertion
Ed	Eye diameter	PFi	Distance between the insertion of the pectoral fin to the bottom of the body
Eh	Distance between the center of the eye to the bottom of the head	PFl	Pectoral fin length
Gl	Total gut length	PFs	Pectoral fin surface
GRL	Gill raker length		

**Table 4:** List of the eight functional morphology traits

Functional traits	Code	Measure	Ecological meaning
Compression index	CI	${\text{Bd}}^{2}/\text{Bl}\times \text{Bw}$	Body transversal or compressed shape
Body section area	BSA	ln((π/4×Bw×Bd)+1)/ln(M+1)	Mass distribution along the body and hydrodynamism
Pectoral fin shape	PFS	${\text{PFl}}^{2}/\text{PFs}$	Propulsion and maneuvrability
Fins area	FA	(2×PFs)+CFs/π/4×Bw×Bd	Acceleration and maneuvrability
Oral gape surface	OGS	Mw×Md×Bw×Bd	Size of food items captured and ability to filter water
Gill raker length	GRL	GRL/Hd	Filtration capacity or gill protection
Gut length	GL	Gl/Bl	Digestibility of food
Eye size	EZ	Ed/Hd	Prey detection

#### 2.3.2 Habitat data

In this study, water temperature (°C), dissolved oxygen (DO, μmol/L), NH_4_–N (mg/L), and total dissolved solids (TDS, g/L) were selected as water quality environmental factors of local habitat. These parameters were determined in situ at each sampling site with a portable multi‐parameter water quality instrument (YSI 6600) twice a month. Water clarity (cm) was detected with a Secchi disk. Precipitation (mm) was selected as the primary climatic factor. Flow velocity (m/s) data were provided by the Pearl River Hydraulic Research Institute.

In this study, river fractal characteristics and coefficients of fluvial facies (C) were selected as river morphological factors of local habitat of fish. Fractal geometry concepts have been widely applied as a tool for describing complex natural phenomena, such as the physics of rivers. The fractal dimension of the river reflects the complexity of the river habitat to some extent. Based on a 10‐km grid map across the Pearl River basin, the river fractal dimensions were computed by the widely used box‐counting method (Liu, Zhang, Shen, Zhao, & Li, 2018). The box sizes used in this study were 10, 8, 6, 4, 2, and 1 km. The river fractals were calculated in ArcGIS 10.2. Coefficient of fluvial facies represents the space and complexity where the fish community can freely move, and is defined as

$$C=\frac{\sqrt{W}}{D}$$,

*W* represents average river width, *D* represents average river depth. Average river depth and width were provided by the Pearl River Hydraulic Research Institute.

### 2.4 Statistical analyses

PCA and PERMANOVA analysis were carried out based on overall morphology in the species and used to test for functional morphological differences among high‐elevation, medium‐elevation, and low‐elevation (Anderson, 2006). To identify how elevation affected specific functional traits, an ANOVA was used to compare functional morphology traits of species between elevation groups (i.e., high versus low‐elevation sites).

Next, multivariate analysis of variance (MANOVA) was used to determine how environmental variables affect the functional morphological variations of each species by using morphological axes generated by geometric functional morphometrics as dependent variables, and local and regional variables as independent variables. In this study, local river physical properties (such as river fractal and river coefficient of fluvial facies) and local water chemical factors (such as water clarity, DO, NH_4_–N, and TDS) were selected as local environment variables. Elevation and temperature were selected as regional factors, which were transformed to three grades as factors and then added as interaction factors into MANOVA. Finally, to further examine which environmental variable that best explains which functional morphological traits and reduce the random effect among species, redundancy analysis (RDA) was performed to test the multiple relationships among fish functional morphology traits and environmental variables. ANOVA permutation tests (replicated randomly 1,000 times) were performed to evaluate the RDA model's performance and significance of constraints.

All analyses were performed with R Software (R Development Core Team, 2013). Variables were considered statistically significant at *p* ˂ 0.05.

## 3 RESULTS

### 3.1 Intra‐species functional morphology difference vs. altitude

**Figure 3:**
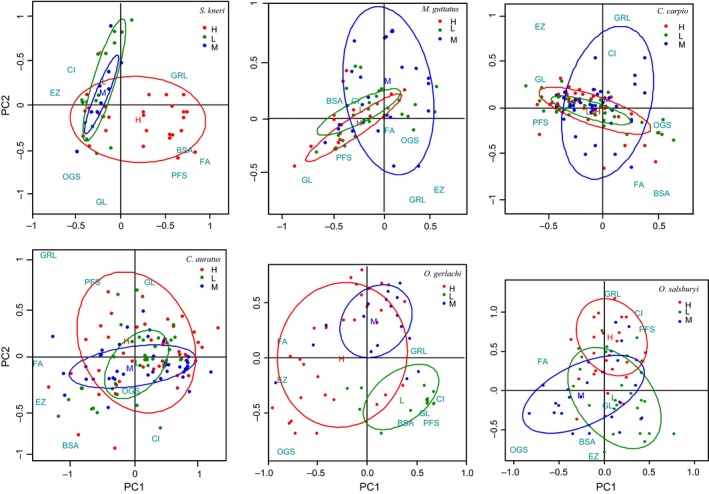
Spatial variations of fish functional morphology using a scatter diagram by elevations

Spatial variations in fish functional morphology are shown in Figure 3. The vast majority of the groups overlapped to some extent. This is because fish functional morphological attributes assume a spatial autocorrelation pattern. Specimens from the high‐elevation group significantly differed from the medium and low‐elevation groups in overall morphology in four species (*p* ˂ 0.05), except the carps, *C. carpio* and *C. auratus*. For the species *O*. *gerlachi*, there were significant differences among the three elevations. For *C*. *carpio* and *C*. *auratus*, there were no differences among the different elevation groups in overall morphology (Figure 3, Table 5).

**Table 5:** Morphological differences of six species at different elevations

Species		H vs. M	H vs. L	M vs. L
*S*. *kneri*	*F*	**4.0642**	**8.0165**	1.6174
	*R* ^*2*^	0.0864	0.1603	0.0496
	*p*	0.016	0.001	0.199
*M*. *guttatus*	*F*	**3.1603**	**5.5802**	1.0975
	*R* ^*2*^	0.0656	0.1311	0.0215
	*p*	0.046	0.01	0.327
*C*. *carpio*	*F*	2.357	0.1457	2.2664
	*R* ^*2*^	0.0293	0.002	0.0318
	*p*	0.105	0.956	0.094
*C*. *auratus*	*F*	0.8708	1.4145	0.3887
	*R* ^*2*^	0.0097	0.019	0.0052
	*p*	0.426	0.212	0.692
*O*. *gerlachi*	*F*	**2.6629**	**8.8198**	**11.007**
	*R* ^*2*^	0.0478	0.1844	0.2684
	*p*	0.046	0.005	0.002
*O*. *salsburyi*	*F*	**3.5188**	**4.7319**	0.4091
	*R* ^*2*^	0.0634	0.0933	0.0075
	*p*	0.042	0.014	0.622

Note

Bold represents a significant difference between elevations (*p* < 0.05), based on a PERMANOVA analysis.

As shown in Figure 3 and Table 6, high‐elevation individuals were more flexible when swimming (reflected in a larger pectoral fin shape (PFS) value) than low‐elevation individuals in four species, except *O*. *gerlachi* and *O*. *salsburyi*. Second, high‐elevation individuals had a more compressed body shape (a smaller compression index (CI) value) than low‐elevation individuals in five species, with the exception of *C*. *carpio*. That is, high‐altitude fish had a narrower body compared to the low‐altitude ones. Third, high‐elevation individuals had a more even distribution of muscle along the body (reflected in a smaller body section area (BSA) value) than low‐elevation individuals in the following four species: *S*. *kneri*,* M*. *guttatus*,* O*. *gerlachi,* and *O*. *salsburyi*. Fourth, high‐elevation individuals showed a stronger ability to filter water (reflected in a larger gill raker length (GRL) value) than low‐elevation individuals in three species: *S*. *kneri*,* O*. *gerlachi,* and *O*. *salsburyi*. In addition, low‐elevation individuals captured larger food sources (reflected in a larger oral gape surface (OGS) value) than high‐elevation individuals in three species: *M*. *guttatus*,* C*. *carpio,* and *O*. *salsburyi*. In addition, small differences in the ability to digest food (gut length [GL]), the visible range of food (eye size [EZ]), and the ability to accelerate when swimming (fins area [FA]) were observed among the three elevations, but these differences were not common among the tested species.

**Table 6:** Functional morphological traits of the six species at different elevations in the Pearl River

Species	Group	Sample size	CI	BSA	PFS	FA	OGS	GRL	GL	EZ
*S*. *kneri*	H	**28**	**5.67** ^**a**^ (0.43)	**53.03** ^**a**^ (3.2)	**33.23** ^**a**^ (6.1)	**0.11** ^**a**^ (0.01)	0.06 (0.007)	**0.27** ^**a**^ (0.01)	0.75 (0.06)	**0.37** ^**a**^ (0.01)
	M	**26**	**6.87** ^**b**^ (0.11)	**76.8** ^**ab**^ (11.5)	**12.06** ^**b**^ (1.03)	**0.07** ^**b**^ (0.005)	0.05 (0.004)	**0.22** ^**b**^ (0.008)	0.76 (0.04)	**0.44** ^**b**^ (0.009)
	L	**27**	**6.85** ^**b**^ (0.12)	**108.4** ^**b**^ (23.9)	**12.19** ^**b**^ (0.9)	**0.09** ^**ab**^ (0.01)	0.05 (0.005)	**0.21** ^**b**^ (0.01)	0.65 (0.03)	**0.39** ^**a**^ (0.013)
*M*. *guttatus*	H	**27**	**1.98** ^**a**^ (0.08)	**105.9** ^**a**^ (23.2)	**3.04** ^**a**^ (0.41)	**0.19** ^**a**^ (0.02)	**0.05** ^**a**^ (0.003)	0.29 (0.01)	1.01 (0.14)	0.35 (0.01)
	M	**26**	**1.89** ^**a**^ (0.07)	**175.8** ^**b**^ (24.9)	**2.07** ^**b**^ (0.31)	**0.25** ^**b**^ (0.02)	**0.06** ^**b**^ (0.005)	0.32 (0.11)	0.84 (0.06)	0.51 (0.12)
	L	**26**	**2.78** ^**b**^ (0.18)	**138.8** ^**ab**^ (13.4)	**1.86** ^**b**^ (0.15)	**0.24** ^**ab**^ (0.01)	**0.05** ^**ab**^ (0.003)	0.29 (0.01)	0.89 (0.04)	0.37 (0.01)
*C*. *carpio*	H	41	6.19 (0.19)	48.4 (10.6)	**10.62** ^**a**^ (2.5)	0.15 (0.011)	**0.02** ^**a**^ (0.001)	0.11 (0.004)	**1.69** ^**a**^ (0.1)	0.29 (0.01)
	M	39	6.45 (0.22)	59.97 (9.5)	**5.93** ^**b**^ (0.35)	0.17 (0.011)	**0.02** ^**a**^ (0.001)	0.14 (0.034)	**1.33** ^**b**^ (0.06)	0.36 (0.072)
	L	32	5.96 (0.14)	44.14 (5.9)	**7.77** ^**ab**^ (0.52)	0.17 (0.013)	**0.04** ^**b**^ (0.001)	0.1 (0.005)	**1.8** ^**a**^ (0.08)	0.3 (0.011)
*C*. *sauratus*	H	**45**	**7.13** ^**a**^ (0.23)	104.9 (13.7)	**10.76** ^**a**^ (2.7)	0.22 (0.01)	0.02 (0.001)	0.23 (0.01)	2.85 (0.23)	0.35 (0.006)
	M	**41**	**8.13** ^**b**^ (0.17)	115.2 (15.6)	**6.4** ^**ab**^ (0.3)	0.24 (0.02)	0.017 (0.001)	0.23 (0.007)	2.55 (0.11)	0.36 (0.009)
	L	**35**	**7.71** ^**b**^ (0.18)	100.1 (7.9)	**5.42** ^**b**^ (0.27)	0.24 (0.01)	0.017 (0.001)	0.22 (0.007)	2.37 (0.15)	0.37 (0.008)
*O*. *gerlachi*	H	**32**	**6.0** ^**a**^ (0.38)	**36.8** ^**a**^ (7.9)	**11.07** ^**ab**^ (2.95)	**0.32** ^**a**^ (0.05)	0.03 (0.004)	**0.054** ^**a**^ (0.003)	**4.78** ^**a**^ (0.57)	**0.34** ^**a**^ (0.01)
	M	**29**	**6.12** ^**a**^ (0.42)	**54.1** ^**ab**^ (18.6)	**7.9** ^**a**^ (0.73)	**0.2** ^**b**^ (0.03)	0.02 (0.002)	**0.06** ^**a**^ (0.004)	**5.75** ^**a**^ (0.46)	**0.31** ^**a**^ (0.01)
	L	**23**	**7.93** ^**b**^ (0.37)	**77.1** ^**b**^ (8.0)	**12.44** ^**b**^ (1.05)	**0.13** ^**b**^ (0.02)	0.03 (0.012)	**0.03** ^**b**^ (0.002)	**8.82** ^**b**^ (0.13)	**0.26** ^**b**^ (0.02)
*O*. *salsburyi*	H	**26**	**5.61** ^**ab**^ (0.16)	**144.5** ^**a**^ (9.2)	**4.46** ^**a**^ (0.27)	0.38 (0.02)	**0. 021** ^**ab**^ (0.001)	**0.073** ^**a**^ (0.006)	4.04 (0.43)	0.32 (0.01)
	M	**26**	**5.48** ^**a**^ (0.12)	**194.7** ^**b**^ (21.5)	**3.5** ^**b**^ (0.22)	0.36 (0.02)	**0.024** ^**a**^ (0.002)	**0.049** ^**b**^ (0.005)	3.4 (0.36)	0.32 (0.007)
	L	**27**	**5.98** ^**b**^ (0.22)	**160.8** ^**ab**^ (10.1)	**4.4** ^**a**^ (0.33)	0.35 (0.03)	**0.019** ^**b**^ (0.001)	**0.049** ^**b**^ (0.006)	3.3 (0.34)	0.33 (0.01)

Note

Functional morphological traits are expressed as means, with standard errors in brackets (SE). Means in bold indicate significant differences between elevations at *p* ˂ 0.05. Abbreviations please refer to Table 4.

### 3.2 Environmental factors distinguish distinct functional morphologies at different elevations

The MANOVA model revealed that fish functional morphological attributes are strongly linked to environmental factors (Table 7). Cumulative % of variances of the functional morphological axes that were more than 60% were selected as dependent variables for MANOVA. Overall, fish functional morphology traits were primarily affected by river physical properties, such as flow velocity, river fractals, and coefficients of fluvial facies (C) in all species. Water clarity was also important factor in functional morphology attributes in four species, with the exception of *C. carpio* and *O*. *salsburyi*. NH_4_–N affected the functional morphological attributes of three species, *C*. *carpio*,* O*. *gerlachi,* and *O*. *salsburyi*. In addition, the effects of the regional variables (elevation and temperature) on functional morphology attributes of fish were stronger than local factors in all species. Especially, the interactive effects of elevation on local environmental factors were significant in three species, *S*. *kneri*,* M*. *guttatus,* and *C*. *carpio* (Table 7).

**Table 7:** Summary of MANOVA and the best explanatory environmental variables for functional morphological axes

	*S*. *kneri*	*M*. *guttatus*	*C*. *carpio*	*C*. *auratus*	*O*. *gerlachi*	*O*. *salsburyi*
Cumulative % of var.	70.7	75.09	78.05	80.38	77.78	76.71
Represents of Axis 1	Fractals, C, Water clarity, Velocity	Fractals, C, Water clarity, Velocity, DO	Fractals, C, Velocity, NH_4_N	Water clarity, Velocity, C, Fractals	Fractals, TDS, C, Velocity, Water clarity, NH_4_N	Fractals, C, NH_4_N, Velocity
Represents of Axis 2	NH_4_N, DO	TDS, NH_4_N	DO, Water clarity,	NH_4_N, DO	DO	Water clarity, DO, TDS
Elevation	**0.0002** ***	**0.0434** *	**0.0463** *	**0.0236** *	**0.0076** **	**0.0027** **
Temperature	**0.0479** *	0.0627	**0.0389** *	**0.0362** *	**0.0145** *	**0.0442** *
Axis 1	**0.0006** ***	**0.0339** *	**0.4739** *	**0.0478** *	**0.0432** *	**0.0329** *
Axis 2	0.2670	0.8365	0.8762	0.5239	**0.0190** *	0.1370
Elevation: Axis 1	**0.0432** *	**0.0368** *	**0.0135** *	0.3286	0.5369	0.1136
Elevation: Axis 2	0.8263	0.2379	0.4578	0.4792	0.5146	0.0693

C represents coefficients of fluvial facies.The effect of environmental factors on the functional morphological axes is expressed as *p*‐values. *p < 0.05; **p < 0.01; ***p < 0.001.

### 3.3 Different environmental factors affect different functional morphologies

**Figure 4:**
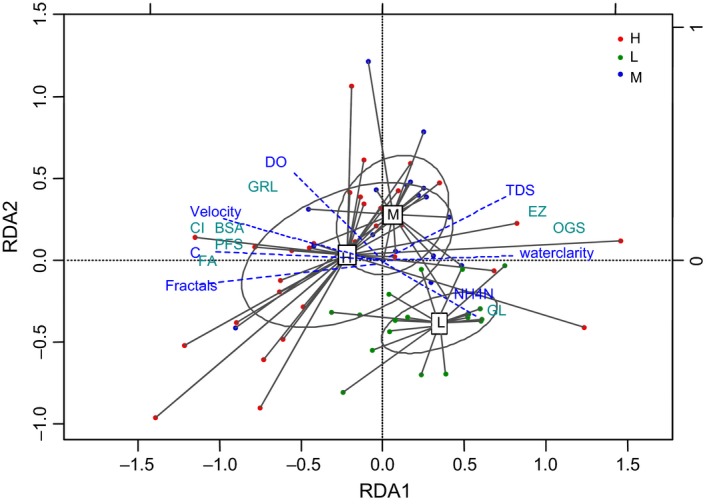
Redundancy analysis triplot showing relationships among fish functional morphology traits and environmental variables (scaling 2). Environmental variables are represented by blue dotted lines; Dark cyan without lines indicates functional morphological traits. DO, dissolved oxygen; NH4–N, ammonia nitrogen content; TDS, total dissolved solids; Fractal, river fractal characteristics; C, Coefficient of fluvial facies

The RDA model further revealed the relationships between fish functional morphology traits and environmental factors (Figure 4). The combined effects of the first two canonical axes explain 82.6% of the total variance of the data. The unadjusted and adjusted *R* ^2^ retrieved from the RDA results were 0.672 and 0.537, respectively, and the *p*‐values (ANOVA test) of the first two canonical axes were sufficiently low to denote a good sample separation along the axis. The RDA triplot (scaling = 2) shows that environments in high‐elevations are characterized by faster flow, increased river fractals, and increased coefficients of fluvial facies (C). Such complex river conditions differ from those at low‐elevations with higher NH_4_–N concentration. The fish body compression index (CI) and body section area (BSA) are related, as are fin area (FA) and pectoral fin shape (PFS), and all were affected by flow velocity, river coefficient of fluvial facie (C), and river fractals. Gill raker length (GRL) was shown to be affected by water quality factors (such as DO), and also associated with flow velocity. Oral gape surface (OGS) and eye size (EZ) are related, and both are affected by water quality factors, water clarity, and TDS. Gut length (GL) was shown to be affected by NH_4_–N concentration (Figure 4).

Overall, functional morphology traits associated with locomotion (i.e., body compression index [CI], body section area [BSA], pectoral fin shape [PFS], and fins area [FA]) were primarily affected by river physical characteristics, such as flow velocity, river fractals, and river coefficient of fluvial facie (C). Functional morphology traits associated with food acquisition (i.e., oral gape surface [OGS], gill raker length [GRL], gut length [GL], and eye size [EZ]) were primarily effected by water chemical factors, such as water clarity, DO, NH4–N concentration, and TDS.

## 4 DISCUSSION

Biotic‐habitat interactions are known to be an important mechanism for species functional phenotype differentiation in organisms. The processes that drive species functional phenotype differentiation have an important role in determining the function differences of an organism in the ecosystem. Understanding the mechanism behind the connection of morphology and ecological performance is central to the study of adaptation and has become one of the main focus of contemporary evolutionary ecology (Siemers & Schnitzler, 2004). Many hypotheses have been raised to explain the species morphology along the environmental gradient, which is one of the most evident features of life on this planet. Bergmann's rule, which posits that within a broadly distributed taxonomic clade, temperature influences body size such that species with a larger size are found in high‐latitude colder environments, while species with a smaller size are found in low‐latitude warmer regions (Bergmann, 1847; Cassey, 2001; Huey, Gilchrist, Carlson, Berrigan, & Serra, 2000). Allen's rule posits that homeothermic animals in hot climates have lower volume‐to‐surface ratios than animals in cold climates due to thermal adaptation (Allen, 1877). Gloger's rule states that within a endothermic species, there is an effect of climate on pigmentation, such that individuals living in more humid habitats tend to be have more heavily pigmented forms than their relatives in regions with higher aridity (Burtt & Ichida, 2004).

The influence of the environment on the organism phenotype will ultimately affect the function of the organism in the ecosystem. Here, we found that altitude also has an important effect on the phenotype of fish, and the traits that varied significantly among elevation groups were body shape, pectoral fin shape, and mass distribution along the body. High‐elevation individuals had a more narrow‐bodied morphology, more flexible maneuvrability when swimming, and more even muscle distribution than low‐elevation individuals. Fish body mass is one of the most important factors influencing energy turnover and consumption during swimming (Boisclair & Tang, 1993). A more uniform muscle distribution along the body enabled some fish to modulate stiffness and sustain higher swimming velocities against the current when exploiting food resources and predation (Gatz, 1979). High‐elevation individuals filter more water, which may also be due to faster water flow velocity in high‐elevation sites, and the amount of water flowing through the gills per hour is increased to get more oxygen for swimming (Wegner, Lai, Bull, & Graham, 2012). Oral gape dimensions were slightly larger in low‐elevation habitats, and may be due to individuals downstream having access to higher quantities of larger food. Where habitat and food size/type co‐vary, food selection matching with mouth size and shape is beneficial to improve foraging efficiency.

Furthermore, it was found that morphology traits associated with locomotion function were primarily affected by river physical characteristics, such as flow velocity, river fractals and river coefficient of fluvial facies. Morphology traits associated with food acquisition function were primarily affected by water chemical factors, such as NH_4_–N concentration, water clarity, DO, and TDS. Our results indicated that the effects of the environment on the biological morphology further affect the functionality of the organism in the ecosystem. River fractals represent the complexity of local habitats (Nestler & Sutton, 2000), while river coefficients of fluvial facies represent the space in which the fish community can freely move, such that the larger the river coefficient of fluvial facies, the bigger the fish activity space; thus, affecting the functional morphology of swimming (Chapman et al., 2015; Langerhans & Reznick, 2010).

Although local environmental factors, such as flow velocity, river fractals and coefficients of fluvial facies, DO, water clarity, NH_4_–N concentration, and TDS, could affect the functional morphological variety, the impact of regional factors (such as elevation and temperature) on the functional morphological variety of fish is greater than the impact of local factors in the Pearl River. This means that the functional morphological traits of fish are affected by the regional environment first, and then by local environmental factors.

Morphology‐habitat associations are common among natural populations, and numerous field studies have shown that stream gradient habitats have an impact on fish morphology have been discussed extensively (Michel, Chien, Beachum, Bennett, & Knouft, 2017). In addition, numerous field studies have shown that stream gradient habitats influence fish morphology across a range of species and many freshwater fish displayed morphologically plastic responses to various habitats (Chapman et al., 2015; Senay, Boisclair, & Peres‐Neto, 2014; Webb, 1984). For example, Crucian carp can alter their body shape in response to different hydraulic conditions and have been reported to develop a shallower body shape when living in water currents (Johansson & Andersson, 2009). Similarly, individuals of *Cyprinella lutrensisi*, a small cyprinid fish, that live in flowing water have shallower bodies than those live in still water (Franssen, 2011). Moreover, fish from sluggish waters generally have laterally compressed, deeper bodies and rounded caudal and paired fins to make the body more flexible for maneuvring and reduce drag (Chapman et al., 2015; Colgate & Lynch, 2004).

The morphological differences between high‐ and low‐altitude fish are the result of phenotypic responses, which led to micro‐evolutionary changes that occurred during the process of adapting to live successfully within local habitats (Bears et al., 2008). Environments that differ at high‐elevations include faster flow and increased river fractals and increased coefficients of fluvial facies. Such complex river conditions differ at low‐elevations with higher NH_4_N concentrations. All of this provides different selection pressures that have an effect on morphological traits, further affecting the function of fish in the ecosystem.

In conclusion, the alterations in body morphology driven by variations in habitat conditions comprise an important functional phenotype differentiation process by which fish adapt to environmental gradients. These variations may in turn further affect the function of those fish in the ecosystem.

## CONFLICT OF INTEREST

None declared.

## AUTHOR CONTRIBUTION

Fangmin Shuai collected samples, performed analyses, and obtained funding. Shixiao Yu designed the experiments. Sovan Lek and Xinhui Li performed analyses. All authors participated in study design and manuscript composition.

## DATA ACCESSIBILITY

Morphological data and environmental data are available at Dryad https://doi.org/10.5061/dryad.5tc2qp0.
